# Unified Protocol for Transdiagnostic Prevention of Depression and Anxiety in Iranian Adolescents: Protocol Development and Initial Outcome Data

**Published:** 2019-04

**Authors:** Abolfazl Mohammadi, Mehdi Soleimani, Mohammad Reza Mohammadi, Imaneh Abasi, Ali Akbar Foroughi

**Affiliations:** 1 Department of Psychiatry, School of Medicine, Tehran University of Medical Sciences, Tehran, Iran.; 2 Department of Clinical Psychology, University of Social Welfare and Rehabilitation Sciences, Tehran, Iran.; 3 Department of Clinical Psychology, Kermanshah University of Medical Sciences, Kermanshah, Iran.

**Keywords:** *Adolescents*, *Anxiety*, *Depression*, *Prevention*, *Transdiagnostic*, * Protocol*

## Abstract

**Objective:** Cognitive-behavioral interventions have been used as effective approaches for the treatment and prevention of depression and anxiety. However, to date, no anxiety and depression prevention guidelines package has been developed for Iranian adolescents. Thus, the purpose of this study was to develop transdiagnostic prevention program of anxiety and depression for Iranian adolescents and to assess the effectiveness of this program in a sample of adolescents.

**Method** **:** Based on evidence-based literatures on CBT interventions, transdiagnostic prevention program was developed and its content and face validity was assessed and established by three clinical psychologies (Ph.D.) and a psychiatrist (child and adolescent postdoctoral). Then, in a semi-experimental design, 62 students were recruited from a school in Tehran by purposive sampling method and were randomly assigned in to experimental (n = 40) and control (n = 22) groups. They participated in 8 sessions of intervention based on the developed program. Revised Child Anxiety and Depression Scale (RCADS)- Child Version and Parent Version- were used to gather the data before, after, and 3 months after intervention.

**Results: **Results of ANCOVA, controlling for the effect of pretest score, showed no significant differences (P>0.05) between experimental and control groups in SAD, panic, MDD, separation anxiety, GAD, OCD, total anxiety, and total anxiety-depression for parent and child in pretest and posttest.

**Conclusion: **Transdiagnostic prevention package for anxiety and depression had no significant effect on reducing anxiety and depression of adolescents. Using an inappropriate measure, difficulties with timing of assessment, and lower severity of pre-intervention anxiety and depression due to universal prevention and sample recruited, might have affected the present findings. Discussion would be clearer and more complete by analyzing follow-up results and education performance in the future.

Mental disorders are the main cause of many worldwide disabilities people suffer from, and when it comes to anxiety and depression, the burden increases significantly (1). Lifetime prevalence of depression and anxiety in adults have been estimated to be 11.2% (2) and 2.4%- 29.8%, respectively (3). In Iran, the prevalence of anxiety and mood disorders have been reported to be 8.35% and 4.29%, respectively (4). In adolescents, lifetime and 12-month prevalence of MDD has been 11.0% and 7.5%, respectively (5), and recent epidemiological studies have reported that the prevalence of anxiety disorders among adolescents ranges from 10% to 31.9% (6). 

In addition to high prevalence of anxiety and depression, they are highly comorbid in adults (7) and adolescents (8). Anxiety and depression comorbidity is one of the strongest predictors of MDD severity (9). 

People with anxiety and depression symptoms are less likely to respond to treatment or report poor treatment outcomes (10, 11). Furthermore, they have earlier age of onset (12). Moreover, the persistence of anxiety disorder is related to depression symptoms (2). Comorbidity is also related to more suicidal ideation, less mental health, and more life dysfunction (13, 14). Anxiety and depression are related to substantial impairment, onset and persistence of other mental disorders, including substance abuse disorders, panic disorder, social anxiety disorder (15-17), and use of primary care services (18). 

Considering the high comorbidity of anxiety and depression and the impairments they cause in various aspects of life, several models and approaches have been developed to explain their etiology and to target common mechanisms responsible for it (19, 20). Transdiagnostic approach to psychopathology and treatment has captured the most attention and has tried to determine shared variables among emotional (anxiety and depression) disorders and to create techniques to improve or modify them in adolescents (21-23). 

Adolescence is a critical period which can make the path to adulthood smooth or unpredictable, with obstacles and mental disorders having the potential to be revealed in this period, which may lead to higher risk of psychological disorders in the future (24). Thus, paying more attention to this generation may be beneficial. Besides, there is emerging evidence on treatment and prevention of mental disorders in adolescents based on recent advances in cognitive behavior therapies, including the transdiagnostic approach and other methods that are similar in process and techniques, such as mindfulness (25) and acceptance (26, 27) interventions.

In recent years, clinical professionals have recognized the importance of focusing on prevention programs, and in line with this effort, some studies have assessed different types of prevention programs for adolescents at risk of anxiety, mood, and other mental disorders and have yielded evidences that support their efficacy and effectiveness (28-30). 

Prevention can reduce the economic and devastating mental burden of mental disorders by targeting some risk factors which are supposed to predispose individuals to long-term psychiatric disorders and it can offer individuals techniques which they can use in time of need. Because anxiety is a precursor of depression and begin before and concurrently in 37% of depressed individuals (31), considering anxiety and depression concurrently in adolescents is extremely important and can yield several clinical implications. On the other hand, the transdiagnostic package could help individuals with wide range of comorbid mental disorders or at risk of developing these disorders by embodying shared mechanisms that affect anxiety and depression and targeting mechanisms which ameliorate or modify these common variables, instead of emphasizing a sole predisposing or maintenance variable responsible for a sole mental disorder such as anxiety and depression. Also, the transdiagnostic package could be used to train a vast majority of clinical professionals who are dealing with anxiety and mood disorders, and this can reduce economic burden of mental health systems. Furthermore, assessing the validity of transdiagnostic package in preventing emotional disorders of adolescents can enhance our knowledge about other utilities of this package. It is also suitable for a wide range of disorders with emotional problems. 

Given the seriousness and sensitivity of teenage years, economical nature and effectiveness of transdiagnostic approach, and probability effect of cultural differences on the effectiveness of different prevention protocols, the aim of this study was to develop a transdiagnostic protocol and to assess the effectiveness of the developed protocol in Iranian adolescents.

## Materials and Methods

***Protocol Development***

Educational guidelines for teachers (instructors) were developed based on the most recent evidence-based findings. This guideline consisted of session by session instruction of implementing anxiety and depression prevention programs based on transdiagnostic components. After initial development of the guideline, it was reviewed by authors and was edited according to instructors’ comments. Furthermore, content validity and face validity of the guideline were evaluated and established by 3 clinical psychologists (Ph.D.) and a psychiatrist (child and adolescent specialist). 

***Protocol Implementation***

After developing the protocol, the anxiety and depression prevention protocol was implemented and assessed by two psychologists (M.Sc. in clinical psychology) in a school in Tehran, capital city of Iran, in a semi-experimental design. The protocol consisted of 8 sessions. The questionnaires were filled in before implementation, after the final session, and three months after the final session. Anxiety and depression prevention protocol is presented in Table 1. According to Cohen formula for sample size, considering the 2 experimental and control groups, and probability of attrition, 40 individuals were selected for each group via purposive sampling method. After initial data cleansing, due to missing data, 18 participants were excluded from the control group and, finally, 62 individuals remained (40 in experimental and 22 in control groups) in the study. Inclusion criteria were age range of 11-18 years and parents’ informed consent for participating in the research. Exclusion criteria were cognitive and mental problems and any mental disorder diagnosis, including anxiety and depression.

After school’s readiness declaration for participating in the study and inclusion and exclusion criteria were assessed by a clinical psychology expert. The individuals were randomly assigned in to control and experimental groups. Revised Child Anxiety and Depression Scale was filled in by participants before and after treatment and at 3 months after treatment. During the follow-up months, there was not any contact between the therapist and participants. 

***Questionnaires***

Revised Child Anxiety and Depression Scale (RCADS) - child version and Revised Child Anxiety and Depression Scale- parent version were used for gathering data.

Revised Child Anxiety and Depression Scale (32): The RCADS is a 74-item questionnaire. It is a self-report scale that assesses a range of psychological disorders in children and adolescents according to DSM-IV criteria, including separation anxiety disorder, social phobia, generalized anxiety disorder, panic disorder, obsessive-compulsive disorder, and major depressive disorder. RCADS is rated on a Likert scale from 0 (never) to 3 (always). Also, it has 2 forms: parent version and child version. This scale has been reported to have acceptable reliability and validity in clinical (33) and non-clinical adolescent samples (32). Internal consistency of the present study is acceptable.

***Clinical Consideration***

APA ethical codes were considered in the process of development and implementation of the prevention protocol. At first, participants were informed about the aims of the research, requirements, and other needed data. They were told they could leave the study whenever they wanted and that participation in the study was voluntarily. Informed consent was obtained and confidentiality was assured. Furthermore, ethical approval was obtained from the Ethical Committee of Tehran University of Medical Sciences (number IR.TUMS.REC.1394.390). 

***Statistical Analysis***

Independent t test and χ2 were used to assess insignificant differences in demographic variables between experimental and control groups. Analysis of covariance (ANCOVA) was used to assess the effectiveness of prevention program.

## Results

A total of 40 and 22 male students were randomly assigned in to experimental and control groups respectively. Demographic information of the participants is shown in Table 2. Study groups were not significantly different in any of demographic variables. Mean and standard deviation of study variables in experimental and control groups are presented in Table 3. Results of ANCOVA controlling the effect of pretest score showed non significant differences (P>0.05) between experimental and control groups in SAD, panic, MDD, separation anxiety, GAD, OCD, total anxiety, and total anxiety-depression for parent and child in pretest and posttest, indicating that our prevention program did not have a significant effect on mental disorders indicators of adolescents (Tables 4 and 5). 

## Discussion

There is growing literature about prevention in internalizing disorders, and in line with this, the present research aimed to develop transdiagnostic anxiety and depression prevention package and to assess the effectiveness of implementing this package in an Iranian adolescent’s sample. Results showed no significant differences between posttest, follow-up, and pretest in any of measured variables, indicating non-significant effect of the developed package. 

Several reasons may be responsible for the low effectiveness of the program, which should be considered carefully in future studies. Trans-agnostic anxiety and depression contents of the prevention package might not have been suitable for adolescents who were not at risk for anxiety and depression. Transdiagnostic contents are different from CBT contents on some levels, as CBT prevention programs had significant effects on reducing depressive symptoms of adolescents at risk of depression in other studies (34, 35). Another reason might have been the competency of the instructors/ teachers for implementing the developed package. Although they were trained to use the package, their training might not have been sufficient. Other reasons could be the questionnaires that were used, which had not been specifically developed for the prevention program. Also, our sample was different from most studies on this subject, and the duration of implementation might not have been enough to obtain adequate results. 

In fact, the present results were not in line with previous findings about the overall but small effectiveness of preventive interventions on anxiety and depression among adolescents (24, 25 and 28). However, a meta-analysis on the effectiveness of depression prevention programs on children and adolescents showed that selective and indicative programs are more effective than universal programs at follow-up because of differences in levels of symptoms in control groups, which is in consistent with our results. In other words, depression and anxiety prevention programs that target selective or indicative adolescents sample may be more practical (36). Furthermore, age and gender could affect the effectiveness of prevention programs. Also, the mechanisms for targeting the prevention of anxiety and depression in adolescents might be different from the mechanisms for targeting the treatment of these disorders, and this should be considered in future studies. Universal prevention programs have some advantages: reducing recruitment, screening, and attrition difficulties; applicability for a range of adolescents with different levels of risks; and reducing the risk of developing later and longer mental disorders and promoting healthy development (37). Beyond these advantages, transdiagnostic prevention programs have some benefits, including fast learning and developing, high capacity to use in groups, and applicability for a range of disorders with shared basics (38, 39). 

**Table 1:** Contents of Anxiety and Depression Prevention Transdiagnostic Protocol for Adolescents

**Sessions**	**Contents**
One	Anxiety definition and its distinction with stress and fear, aspects of anxiety (cognitive, behavioral, and somatic), and its reciprocal relations
Two	Explaining physical basis of anxiety and depression, introducing vicious cycle between brain and physical aspect of stress, introducing and practicing body and muscle awareness, and mindful belly breathing techniques
Three	Introducing concepts of mindfulness and its aspects, practicing eating raisins as a mindfulness technique, introducing decentering from thoughts and emotions, and practicing its technique
Four	Explaining the role of thoughts in emotions and behaviors, introducing negative automatic thoughts and ABCDE model, introducing cognitive restructuring and cognitive errors
Five	Introducing awareness without judgment and its aspects, introducing response without reacting, practicing 3-minute breathing practice
Six	Introducing behavioral activation, explaining the advantages of behavioral activation planning for pleasurable and constructive activities
Seven	Explaining the role of avoidance in sadness, anxiety, and worry
Eight	Introducing problem-solving technique and its steps

**Table 2:** Demographic Information of Participants of Experimental and Control Groups

**Variable**	**Experimental Group**			**Control Group**			**t**	***χ*** ^2^
	**M (SD)**	**N**	**%**	**M (SD)**	**N**	**%**		
Parent Gender								1.37
Male		9	22.5		8	36.4		
Female		31	77.5		14	63.6		
Occupation								0.17
Housekeeper		11	27.5		5	22.7		
Employed		29	72.5		17	77.3		
Education								2.73
Diploma		8	20.0		1	4.5		
Higher than Diploma		32	80.0		21	95.5		
Parent age	43.4 (3.9)			41.3(4.2)			1.99	
Students age	12.7 (0.6)			12.9(0.4)			-0.18	

Note: None of the variables was significant at P<0.05.

**Table 3:** Mean (Standard Deviation) of Study Variables in Experimental and Control Groups

**Experimental Group**				**Control Group**		
**Variable**	**Pretest**	**Post-test**	**Follow-up**	**Pretest**	**Posttest**	**Follow-Up**
SAD- parent	6.3 (5.4)	5.9 (4.6)	5.6 (4.1)	6.2 (2.9)	5.4 (2.1)	7.0 (5.1)
Panic-parent	1.9 (3.1)	1.6 (1.9)	3.8 (3.8)	1.8 (1.3)	1.7 (1.3)	5.2 (3.6)
MDD-parent	3.1 (3.4)	2.9 (2.1)	4.2 (3.3)	2.2 (2.8)	2.3 (2.7)	5.1 (3.4)
Separation-parent	3.1 (3.6)	2.7 (3.1)	2.9 (3.0)	1.8 (1.8)	1.8 (1.7)	3.5 (2.8)
GAD-parent	3.2 (3.5)	2.9 (2.8)	2.9 (2.7)	2.7 (2.0)	2.5 (1.8)	4.4 (3.3)
OCD-parent	2.2 (2.9)	2.1 (2.6)	3.1 (3.1)	2.0 (2.5)	1.7 (2.1)	3.4 (2.5)
Whole anxiety-parent	16.7(15.7)	15.2(12.9)	18.4 (15.2)	14.4 (7.7)	13.3 (6.4)	23.5(14.5)
Anxious-depression parent	19.9(18.1)	18.1(14.1)	22.6(17.7)	16.6(9.1)	15.5(8.0)	28.7(17.1)
SAD- child	11.5(5.9)	9.5(4.7)	8.2(4.8)	9.7(5.9)	7.9(4.5)	7.4(4.9)
Panic-child	5.8(4.6)	5.1(4.1)	4.7(4.0)	5.7(4.0)	5.2(3.2)	5.3(4.0)
MDD-child	5.8(4.3)	5.3(3.9)	4.6(3.8)	4.4(3.9)	4.1(3.3)	3.9(3.5)
Separation-child	3.4(3.2)	3.4(3.2)	2.8(2.5)	3.3(2.5)	3.2(2.3)	2.4(2.9)
GAD-child	6.5(3.5)	5.7(2.9)	4.9(2.7)	5.6(3.9)	5.3(3.8)	4.8(3.2)
OCD-child	5.6(3.8)	4.7(2.7)	3.9(2.9)	5.4(3.2)	4.9(2.7)	4.4(2.5)
Whole anxiety-child	32.7(17.3)	28.4(14.2)	24.4(14.3)	30.1(16.7)	26.6(14.6)	24.8(15.2)
Anxious-depression child	38.5(19.6)	33.8(16.5)	29.0(16.8)	34.5(20.1)	30.7(17.3)	28.7(18.3)

**Table 4:** ANCOVA Results of Study Variables in Posttest Assessments of Experimental and Control Groups

**Variable**	**Homogeneity of Variances**	**Pretest Effect**	**Group Effect**	**Partial η2**
SAD- parent	F(1, 60)=0.1	F(1, 59)=921.8	F(1, 59)=0.2	0.02
SAD- child	F(1, 60)=0.2	F(1, 59)=924.4	F(1, 59)=0.5	0.01
Panic-parent	F(1, 60)=0.3	F(1, 59)=237.8	F(1, 59)=1.2	0.02
Panic-child	F(1, 60)=0.0	F(1, 59)=1315.4	F(1, 59)=0.5	0.01
MDD-parent	F(1, 60)=0.2	F(1, 59)=164.2	F(1, 59)=0.0	0.00
MDD-child	F(1, 60)=0.0	F(1, 59)=1270.4	F(1, 59)=0.3	0.00
Separation-parent	F(1, 60)=1.1	F(1, 59)=853.2	F(1, 59)=1.7	0.03
Separation-child	F(1, 60)=0.6	F(1, 59)=173.8	F(1, 59)=0.0	0.00
GAD-parent	F(1, 60)=7.5	F(1, 59)=860.4	F(1, 59)=0.0	0.00
GAD-child	F(1, 60)=0.8	F(1, 59)=931.1	F(1, 59)=2.7	0.04
OCD-parent	F(1, 60)=1.2	F(1, 59)=1281.6	F(1, 59)=1.9	0.03
OCD-child	F(1, 60)=2.1	F(1, 59)=303.0	F(1, 59)=1.9	0.03
Whole anxiety-parent	F(1, 60)=5.7	F(1, 59)=2692.4	F(1, 59)=0.0	0.00
Whole anxiety-child	F(1, 60)=0.3	F(1, 59)=1919.0	F(1, 59)=0.4	0.03
Anxious-depression parent	F(1, 60)=3.8	F(1, 59)=1431.1	F(1, 59)=0.0	0.00
Anxious-depression child	F(1, 60)=0.0	F(1, 59)=2586.7	F(1, 59)=0.3	0.00

**Table 5:** ANCOVA Results of Study Variables at Follow-Up Assessments of Experimantal and Control Groups

**Variable**	**Homogeneity of Variances**	**Pretest Effect**	**Group Effect**	**Partial η2**
SAD- parent	F(1, 60)=1.6	F(1, 59)=1.5	F(1, 59)=1.5	0.02
SAD- child	F(1, 60)=4.2	F(1, 59)=84.1	F(1, 59)=0.2	0.00
Panic-parent	F(1, 60)=0.0	F(1, 59)=0.1	F(1, 59)=1.9	0.03
Panic-child	F(1, 60)=1.2	F(1, 59)=241.6	F(1, 59)=2.1	0.03
MDD-parent	F(1, 60)=0.0	F(1, 59)=4.5	F(1, 59)=1.8	0.03
MDD-child	F(1, 60)=2.4	F(1, 59)=246.7	F(1, 59)=0.5	0.01
Separation-parent	F(1, 60)=0.3	F(1, 59)=0.6	F(1, 59)=0.8	0.01
Separation-child	F(1, 60)=1.1	F(1, 59)=192.1	F(1, 59)=0.4	0.01
GAD-parent	F(1, 60)=1.9	F(1, 59)=1.5	F(1, 59)=3.5	0.06
GAD-child	F(1, 60)=0.3	F(1, 59)=162.7	F(1, 59)=1.5	0.04
OCD-parent	F(1, 60)=0.8	F(1, 59)=3.2	F(1, 59)=0.2	0.00
OCD-child	F(1, 60)=3.7	F(1, 59)=120.2	F(1, 59)=2.1	0.03
Whole anxiety-parent	F(1, 60)=0.1	F(1, 59)=0.7	F(1, 59)=1.8	0.03
Whole anxiety-child	F(1.60)=4.2	F(1, 59)=178.5	F(1, 59)=1.5	0.02
Anxious-depression parent	F(1, 60)=0.1	F(1, 59)=0.7	F(1, 59)=1.9	0.17
Anxious-depression child	F(1, 60)=4.8	F(1, 59)=200.5	F(1, 59)=1.6	0.03

## Limitations

The present findings should be interpreted cautiously in light of some limitations. First, the measure used for assessing the effectiveness of the package was limited to clinical symptoms and as sample was general, this measure might have not assessed some moderating and mediating variables as coping strategies and happiness. Second, assessment was done when students were under stress, so it is recommended to replicate this study in another time by controlling confounding variables such as stress. Furthermore, other confounding factors should be controlled and considered in future researches. Third, the sample was male and the results might have been different in females as depression and at some points anxiety psychopathologies are different in males and females. 

It is recommended to develop a prevention package considering certain risk factors moderating or mediating the relationship between anxiety and depression and the intervention based on multiple components of emotional symptoms. Moreover, it is suggested to target parenting behaviors that are related to anxiety and depression symptoms of adolescents in future prevention program development based on theories, preferably considering contextual implications.

## Conclusion

The present findings shed light on future progression in developing transdiagnostic prevention programs for adolescents. Also, in line with previous findings, this study suggests that prevention programs may be more applicable for selective and indicative prevention unless some critical changes are made in prevention packages based on valid literatures and proposed suggestions.
